# Challenges in Diagnosing and Managing Hurler Syndrome: A Case Report

**DOI:** 10.7759/cureus.67056

**Published:** 2024-08-17

**Authors:** Lovett S Achiatar, Hussain B Hazoor, Rahul Adwani, Vaishvik K Patel, Ali Gul

**Affiliations:** 1 Acute Medicine, Medway Maritime Hospital, Kent, GBR; 2 Internal Medicine, Pak Red Crescent Medical and Dental College, Lahore, PAK; 3 Internal Medicine, Dow University of Health Sciences, Karachi, PAK; 4 Internal Medicine, St. George’s University, West Indies, GRD; 5 General Surgery, Nishtar Medical University, Multan, PAK

**Keywords:** developmental delays, glycosaminoglycan accumulation, alpha-l-iduronidase deficiency, mucopolysaccharidosis type i, hurler syndrome

## Abstract

This case report details a 12-year-old male diagnosed with Hurler syndrome, a rare autosomal recessive disorder caused by a deficiency in the enzyme alpha-L-iduronidase. The patient exhibited typical symptoms, including developmental delays, ocular clouding, and distinctive skeletal deformities, along with mild cognitive abnormalities. Despite the presence of traditional clinical signs and elevated urine heparin and dermatan sulfate levels confirming the diagnosis, access to advanced treatments such as enzyme replacement therapy was severely limited due to socioeconomic constraints and a lack of diagnostic facilities in the region. This case highlights the critical need for accessible diagnostic and treatment options in resource-limited settings and underscores the importance of ethical decision-making in managing rare genetic disorders. The report advocates for a multidisciplinary approach to enhance outcomes for patients with Hurler syndrome.

## Introduction

Hurler syndrome, also known as mucopolysaccharidosis type I (MPS I), is a rare autosomal recessive disorder first identified by Gertrud Hurler in 1919. It results from a deficiency of the enzyme alpha-L-iduronidase, essential for the degradation of glycosaminoglycans (GAGs) such as heparan sulfate and dermatan sulfate. This enzyme deficiency leads to the accumulation of GAGs in lysosomes, causing severe and progressive symptoms. Clinically, patients exhibit developmental delays, significant organomegaly, and distinctive facial dysmorphisms, including macrocephaly, widely spaced eyes, and coarse facial features [1]. Skeletal abnormalities, such as short stature and distinctive hand and rib deformities, further characterize the disease [2].

Despite advancements in diagnostic techniques, such as enzyme assays, and therapeutic interventions like enzyme replacement therapy (ERT) and hematopoietic stem cell transplantation (HSCT), managing Hurler syndrome remains challenging. These challenges are particularly acute in resource-limited settings, where access to advanced diagnostics and treatments may be restricted. Effective management requires a comprehensive, multidisciplinary approach that includes genetic counseling to address hereditary implications and coordinated medical care to manage the diverse systemic manifestations. Such an approach aims to improve patient outcomes and provide holistic support to affected families, underscoring the need for tailored care strategies in treating rare genetic disorders [3].

This case report aims to delineate the clinical presentation and management challenges of Hurler syndrome in a resource-constrained environment. It seeks to illuminate both typical and atypical disease manifestations, highlight diagnostic difficulties due to limited access to specialized tests, and explore treatment options amid socioeconomic constraints. Through this report, we intend to enhance understanding of the ethical and practical realities of treating rare genetic disorders and contribute to the literature by detailing a management strategy that addresses both symptomatic and enzymatic deficiencies, thereby aiding clinicians in similar contexts.

## Case presentation

A 12-year-old male patient was referred to a tertiary care hospital, presenting with respiratory distress, cough, and abdominal distention. The parents reported that his developmental milestones began to slow down around age two, noting difficulties in standing and progressive cloudiness in the corneas, leading to greater vision loss in the left eye compared to the right. No hearing loss was reported. The patient was observed to have an enlarged tongue, which impaired his speech. Unlike the severe mental retardation typically seen in similar cases, he exhibited some cognitive impairments but had been attending school from an early age. The patient’s family history was significant for the consanguineous marriage between his parents (first cousins), and he was born at home through a spontaneous vaginal delivery assisted by a midwife. He was the eldest of four siblings who did not exhibit any apparent mental or physical abnormalities, and the family had no known history of similar anomalies.

**Figure 1:**
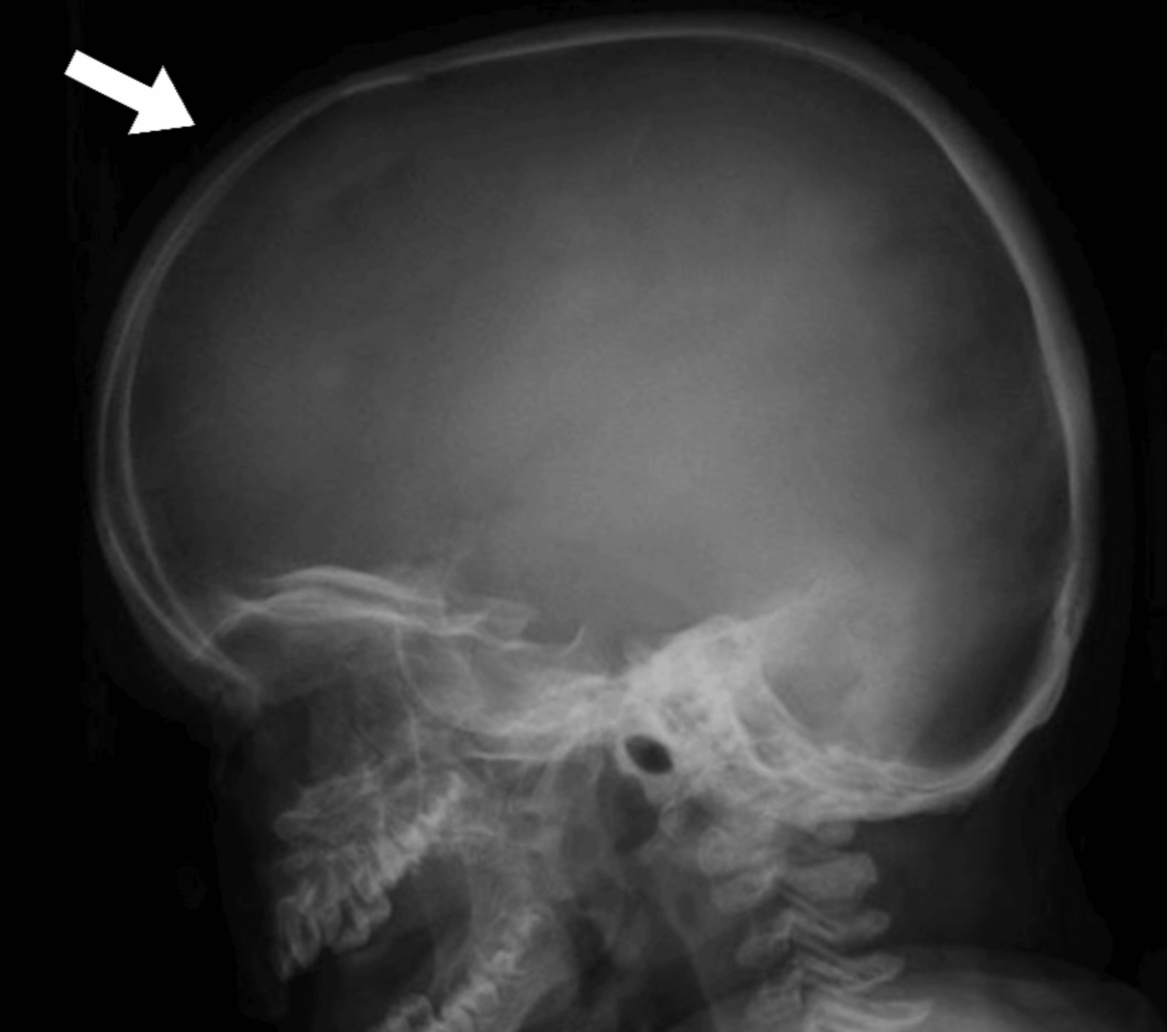
Skull X-ray showing macrocephaly, with the arrow highlighting the skull

**Figure 2:**
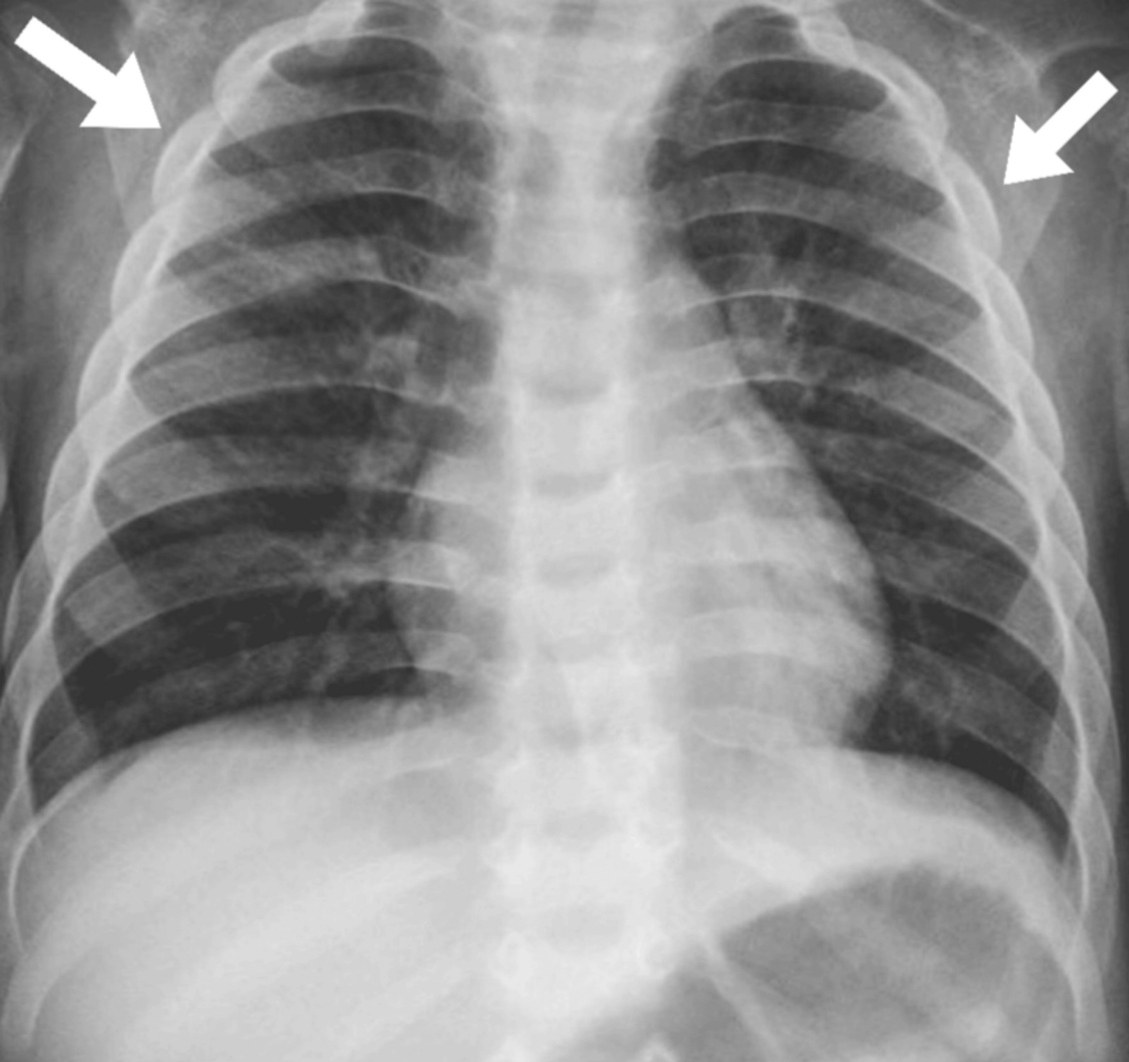
Chest X-ray displaying broad, “oar”-shaped ribs, as indicated by the arrows

**Figure 3:**
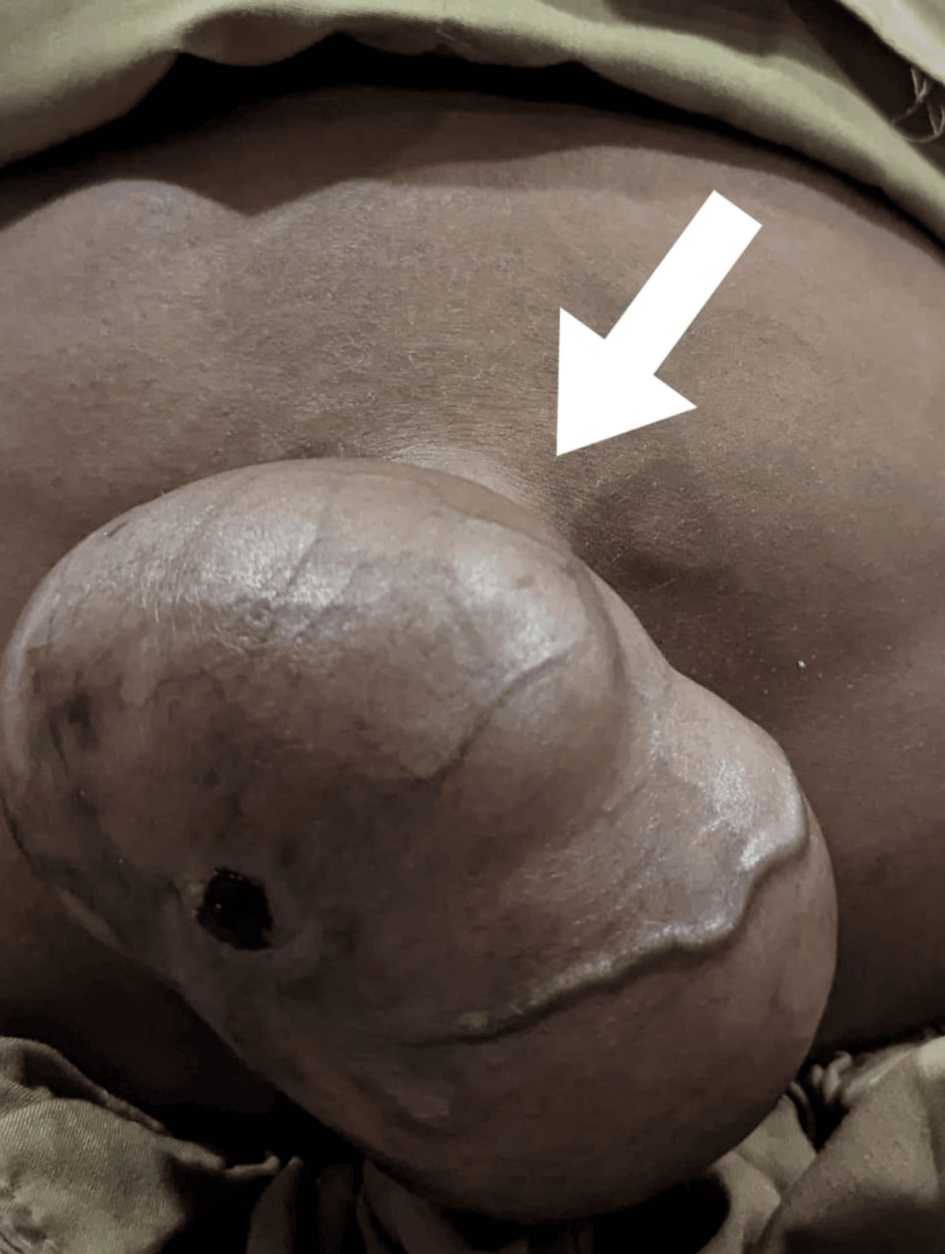
Abdominal examination showing a prominent umbilical hernia (arrow), a common feature of Hurler syndrome

During the clinical examination, the patient displayed coarse facial features, including widely spaced eye sockets with protruding eyes, enlarged lips, and a short neck. The patient exhibited macrocephaly, as shown in Figure 1. The chest X-ray indicated broad, “oar”-shaped ribs, as illustrated in Figure 2. The examination also revealed severe hepatosplenomegaly and an umbilical hernia, as shown in Figure 3.

A provisional diagnosis of MPS I was considered based on a thorough review of the clinical history, detailed examination findings, and characteristic skeletal abnormalities observed in radiographic imaging. The presence of mild cognitive impairments further supported a specific diagnosis of Hurler syndrome. To confirm the diagnosis, extensive biochemical testing was performed, including urine analysis for increased levels of heparan sulfate and dermatan sulfate. The results revealed heparan sulfate levels at 2.5 times the normal upper limit and dermatan sulfate levels at three times the normal upper limit, both of which are markedly elevated and indicative of Hurler syndrome. Additionally, the baseline blood workup showed mild anemia, normal white blood cell and platelet counts, and elevated liver function tests, suggesting liver involvement. All relevant data are provided in Table 1.

**Table 1:** Laboratory test results of the patient

Test	Result	Reference range	Units
Urinary heparan sulfate	25 mg/mmol creatinine	≤15 mg/mmol creatinine	mg/mmol creatinine
Urinary dermatan sulfate	30 mg/mmol creatinine	≤15 mg/mmol creatinine	mg/mmol creatinine
Alpha-L-iduronidase activity	Decreased	Normal enzyme activity	U/L
Hemoglobin	10.5	13.8-17.2	g/dL
White blood cell count	8,000	4,500-11,000	cells/µL
Platelet count	220,000	150,000-450,000	cells/µL
Aspartate aminotransferase	80	10-40	U/L
Alanine aminotransferase	95	7-56	U/L
Alkaline phosphatase	250	44-147	U/L

Several other conditions were considered during the diagnostic process, given the patient’s symptoms and physical findings. These included Hunter syndrome and Maroteaux-Lamy syndrome, which share some phenotypic features with Hurler syndrome, such as organomegaly, facial dysmorphia, and developmental delays. However, Hunter syndrome typically presents with clearer corneas and less severe developmental delays, while Maroteaux-Lamy syndrome usually does not involve cognitive impairment. Specific enzyme assays were conducted to rule out these conditions by confirming the absence of the enzyme deficiencies characteristic of these other mucopolysaccharidoses and verifying the deficiency in alpha-L-iduronidase, thereby confirming the diagnosis of Hurler syndrome.

The management of Hurler syndrome in this patient was multifaceted, focusing on both symptomatic relief and attempts to address the underlying disease process. ERT with laronidase, the standard treatment for Hurler syndrome, was considered to alleviate non-neurological manifestations and improve quality of life. However, the parents hesitated to proceed with this long-term treatment due to financial constraints. Symptomatic treatment included managing bronchopneumonia with appropriate antibiotics and respiratory support to relieve immediate respiratory distress. Regular physical therapy was recommended to address mobility issues caused by skeletal abnormalities. Pain management was tailored to the patient’s needs, incorporating both pharmacologic and non-pharmacologic measures to alleviate discomfort related to organomegaly and joint stiffness. The healthcare team emphasized the importance of regular follow-up visits to monitor disease progression and adjust the treatment plan as necessary. Discussions with a genetic counselor were offered to help the family understand the hereditary nature of the condition and discuss potential implications for future family planning.

## Discussion

Hurler syndrome, or MPS I, is a severe lysosomal storage disorder caused by mutations in the IDUA gene, which encodes the enzyme alpha-L-iduronidase. This enzyme deficiency leads to GAG accumulation in tissues, causing various systemic impairments. The genetic basis of Hurler syndrome indicates an autosomal recessive inheritance pattern, underscoring the importance of genetic counseling for at-risk families [4].

Epidemiologically, Hurler syndrome is rare, with an estimated occurrence of 1 in 100,000 live births globally. However, specific populations may exhibit higher rates due to founder effects or genetic drift [5]. Studies have indicated variable prevalence rates in different geographic regions and among distinct ethnic groups. These epidemiological insights are crucial for developing targeted genetic screening programs and enhancing awareness in primary care settings, which can lead to earlier diagnosis and intervention.

The pathophysiology of Hurler syndrome is closely linked to the deficiency of alpha-L-iduronidase, which is essential for the breakdown of dermatan sulfate and heparan sulfate, two major types of GAGs [6]. The pathological accumulation of these substances in lysosomes disrupts normal cellular function and manifests in multiple organ systems, leading to the characteristic features of the syndrome, including skeletal deformities, coarse facial features, organomegaly, and neurological impairments.

Clinically, the manifestations of Hurler syndrome may vary widely, presenting a spectrum from mild to severe. The patient exhibited classical features such as short stature, coarse facies, corneal clouding, and skeletal deformities, including “bullet-shaped” metacarpals and “oar-shaped” ribs. Notably, the cognitive impairments in our patient were milder than typically observed, which may suggest variability in the expression of the disease or differences in the residual activity of alpha-L-iduronidase. Comparing these manifestations with typical presentations in the literature reveals that while some features are nearly universal among patients, the severity and progression of symptoms can differ significantly. This variability underscores the complexity of MPS I, its management, and the challenges in predicting prognosis.

Diagnosing Hurler syndrome is particularly challenging in resource-limited regions due to the nonspecific nature of early symptoms like developmental delays and recurrent infections, coupled with a shortage of specialized diagnostic facilities, often leading to delayed recognition until severe manifestations emerge. However, recent advancements have significantly improved early detection, including identifying sensitive biomarkers in bodily fluids indicative of abnormal GAG metabolism preceding overt symptoms and the increased utilization of non-invasive imaging modalities like MRI and ultrasound to assess characteristic organ and skeletal abnormalities. Moreover, genetic testing is pivotal in a definitive diagnosis by identifying IDUA gene mutations, enabling carrier screening and prenatal testing, profoundly impacting family planning, and facilitating timely intervention strategies [7].

The primary therapeutic approaches for Hurler syndrome encompass HSCT and ERT. HSCT, involving stem cell transplantation to restore the missing enzyme production, offers a potential cure if implemented early in the disease course, albeit with risks such as graft-versus-host disease and donor compatibility constraints. ERT, typically with laronidase (aldurazyme), replaces the deficient alpha-L-iduronidase enzyme, ameliorating various physical manifestations like hepatosplenomegaly and pulmonary dysfunction; however, its efficacy in addressing neurological outcomes is limited due to its inability to cross the blood-brain barrier [8]. Recent ERT advancements focus on enhancing enzyme delivery to the brain and developing formulations with improved tissue penetration. At the same time, ongoing clinical trials explore gene therapy as a prospective long-term solution, aiming to correct the underlying genetic defect [9].

The management of a rare and severe condition like Hurler syndrome presents unique ethical difficulties, particularly in resource-constrained settings. These difficulties revolve around the judicious allocation of scarce resources, decision-making regarding access to expensive treatments like ERT and HSCT, and the ethical handling of cases where optimal treatment is financially prohibitive. These scenarios necessitate a delicate balance between beneficence, justice, and respect for people, ensuring equitable access to care regardless of socioeconomic status. Socioeconomic barriers significantly impede access to advanced therapies, as the high costs of long-term treatments like ERT are often not covered by insurance in many countries, rendering them inaccessible for numerous families. Health policy reforms incorporating coverage for rare disease treatments and patient support programs subsidizing drug costs can play a pivotal role in improving treatment accessibility. Moreover, establishing national registries and specialized treatment centers can streamline care and reduce costs through centralized expertise.

The decision-making process regarding the patient’s treatment was heavily influenced by socioeconomic factors. Despite the availability of potentially life-extending treatments like ERT, the family’s financial constraints led them to decline these options. This scenario highlights the profound impact of socioeconomic factors on health outcomes and underscores the need for healthcare systems to provide robust support mechanisms for families grappling with rare diseases [10].

The management of Hurler syndrome remains an area requiring extensive research to address significant gaps. Developing therapeutics capable of crossing the blood-brain barrier to treat neurological manifestations is imperative. Gene therapy advancements hold promise for a potential one-time curative approach, superseding ongoing treatments. Furthermore, enhancing diagnostic precision, mainly through newborn screening programs, could facilitate early intervention, profoundly impacting disease outcomes. Future studies should evaluate the long-term efficacy and identify late-onset complications of current treatments. Clinical trials exploring advanced gene-editing technologies like CRISPR for direct genetic correction could pave the way for groundbreaking treatment modalities. By addressing these research avenues and developing more effective and accessible treatments, the medical community can enhance Hurler syndrome patients' prognosis and quality of life, ensuring equitable access to cutting-edge medical advancements [11].

## Conclusions

This case report of a 12-year-old patient with Hurler syndrome highlights the significant challenges and complexities of diagnosing and managing rare genetic disorders in resource-limited settings. The patient’s presentation, featuring classic symptoms of MPS I along with mild cognitive impairments, underscores the variability in disease manifestation and the necessity of a comprehensive diagnostic approach. Although Hurler syndrome was confirmed through biochemical markers, limitations in local testing facilities and socioeconomic barriers significantly impacted the available treatment options. This case exemplifies the need for improved access to advanced therapies and genetic counseling and underscores the role of healthcare policy in supporting families affected by rare diseases. Overall, it advocates for a multidisciplinary approach to enhance patient outcomes and emphasizes the importance of ethical considerations in managing expensive and long-term treatments for rare genetic conditions.

## References

[1] Sakuru R, Bollu PC (2024). Hurler syndrome. StatPearls [Internet].

[2] Khalid N, Abdullah M, Awais AB, Hassan M, Muhammad A (2023). Hurler syndrome (mucopolysaccharidosis type 1): a case report. Cureus.

[3] Lamichhane S, Sapkota A, Sapkota S, Adhikari N, Aryal S, Adhikari P (2024). Mucopolysaccharidosis type I Hurler-Scheie syndrome: a case report. Ann Med Surg (Lond).

[4] Machnikowska-Sokołowska M, Myszczuk A, Wieszała E, Wieja-Błach D, Jamroz E, Paprocka J (2023). Mucopolysaccharidosis type 1 among children—neuroradiological perspective based on single centre experience and literature review. Metabolites.

[5] Çelik B, Tomatsu SC, Tomatsu S, Khan SA (2021). Epidemiology of mucopolysaccharidoses update. Diagnostics (Basel).

[6] Hinek A, Wilson SE (2000). Impaired elastogenesis in Hurler disease: dermatan sulfate accumulation linked to deficiency in elastin-binding protein and elastic fiber assembly. Am J Pathol.

[7] Hampe CS, Eisengart JB, Lund TC, Orchard PJ, Swietlicka M, Wesley J, McIvor RS (2020). Mucopolysaccharidosis type I: a review of the natural history and molecular pathology. Cells.

[8] Ghosh A, Miller W, Orchard PJ (2016). Enzyme replacement therapy prior to haematopoietic stem cell transplantation in mucopolysaccharidosis type I: 10 year combined experience of 2 centres. Mol Genet Metab.

[9] Eisengart JB, Rudser KD, Xue Y (2018). Long-term outcomes of systemic therapies for Hurler syndrome: an international multicenter comparison. Genet Med.

[10] Hajjaj FM, Salek MS, Basra MK, Finlay AY (2010). Non-clinical influences on clinical decision-making: a major challenge to evidence-based practice. J R Soc Med.

[11] Kiely BT, Kohler JL, Coletti HY, Poe MD, Escolar ML (2017). Early disease progression of Hurler syndrome. Orphanet J Rare Dis.

